# Design, Synthesis and Evaluation of New Bioactive Oxadiazole Derivatives as Anticancer Agents Targeting Bcl-2

**DOI:** 10.3390/ijms21238980

**Published:** 2020-11-26

**Authors:** Rania Hamdy, Samia A. Elseginy, Noha I. Ziedan, Mohamed El-Sadek, Elsaid Lashin, Arwyn T. Jones, Andrew D. Westwell

**Affiliations:** 1 School of Pharmacy and Pharmaceutical Sciences, Cardiff University, Redwood Building, King Edward VII Avenue, Cardiff CF10 3NB, Wales, UK; samiaaliph@gmail.com (S.A.E.); n.ziedan@chester.ac.uk (N.I.Z.); JonesAT@cardiff.ac.uk (A.T.J.); 2 Faculty of Pharmacy, Zagazig University, Zagazig 44519, Egypt; m.elhussenyelsadek@yahoo.com (M.E.-S.); slashine@yahoo.com (E.L.); 3 Research Institute for Medical and Health Sciences, University of Sharjah, P.O. Box 27272, Sharjah, UAE; 4 Green Chemistry Department, Chemical Industries Research Division, National Research Center, Cairo, Egypt; 5 School of Biochemistry, University of Bristol, University Walk, Bristol BS8 1TD, UK; 6 Department of Mathematical and Physical Sciences, University of Chester, Chester CH2 4NU, UK

**Keywords:** oxadiazole, indole, aromatic heterocycle, anticancer, Bcl-2 inhibitor, chemical synthesis, molecular modeling, apoptosis

## Abstract

A series of 2-(1*H*-indol-3-yl)-5-substituted-1,3,4-oxadiazoles, **4a–m,** were designed, synthesized and tested in vitro as potential pro-apoptotic Bcl-2 inhibitory anticancer agents based on our previously reported hit compounds. Synthesis of the target 1,3,4-oxadiazoles was readily accomplished through a cyclization reaction of indole carboxylic acid hydrazide **2** with substituted carboxylic acid derivatives **3a–m** in the presence of phosphorus oxychloride. New compounds **4a–m** showed a range of IC_50_ values concentrated in the low micromolar range selectively in Bcl-2 positive human cancer cell lines. The most potent candidate 4-trifluoromethyl substituted analogue **4j** showed selective IC_50_ values of 0.52–0.88 μM against Bcl-2 expressing cell lines with no inhibitory effects in the Bcl-2 negative cell line. Moreover, **4j** showed binding that was two-fold more potent than the positive control gossypol in the Bcl-2 ELISA binding affinity assay. Molecular modeling studies helped to further rationalize anti-apoptotic Bcl-2 binding and identified compound **4j** as a candidate with drug-like properties for further investigation as a selective Bcl-2 inhibitory anticancer agent.

## 1. Introduction

**Figure 1:**
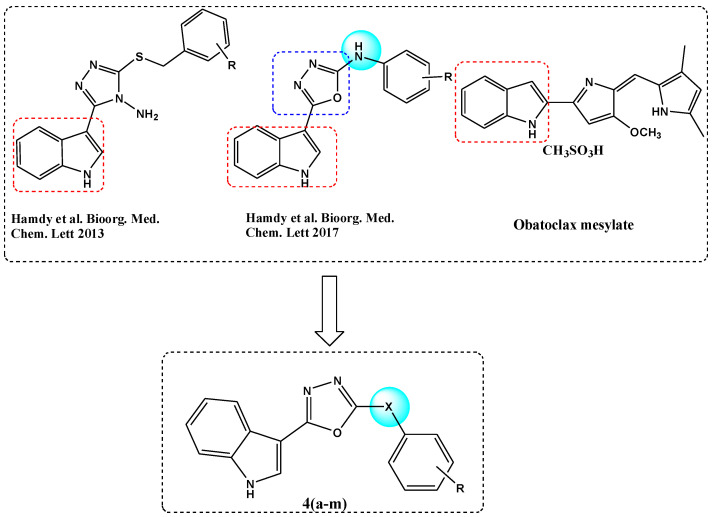
Rational design of novel indole-based oxadiazoles **4a–m.**

The archetypal member of the Bcl-2 anti-apoptotic gatekeeper protein family is a well-studied drug target [1], validated in recent years by the clinical approval of Venetoclax (ABT-199) for the treatment of chronic lymphocytic leukemia, small lymphocytic lymphoma and acute myeloid leukemia [2]. As a Bcl-2 homology domain (BH3) mimetic, Venetoclax represented an important development within the area of protein–protein interaction (PPI) inhibitors. As a target class, PPI inhibitors had previously been thought to be rather intractable drug targets [3]; however, Venetoclax represents an early example of a PPI inhibitor targeting a hot-spot domain that was validated in clinical trials. Other Bcl-2 inhibitory agents that have been studied in cancer clinical trials include obatoclax mesylate [4], an indole-based Bcl-2 inhibitor that received orphan drug designation in the US, in 2004, for the treatment of chronic lymphocytic leukemia (Figure 1). The relatively simple heterocyclic structure of obatoclax has provided inspiration for our own studies on a range of indole-azole-linked Bcl-2 inhibitory anticancer agents. Previously we reported a series of indole-based oxadiazole amines [5,6] and indole-based triazole amines [7] as Bcl-2 inhibitors (Figure 1). Within this series, we found that the oxadiazoles were more active than the corresponding triazoles [7], with sub-micromolar activity found for the most active indole-oxadiazole amine series members in the Bcl-2 ELISA binding assay. In addition, we discovered new quinolinyl-based oxadiazoles and triazoles as potent Bcl-2 inhibitory anticancer compounds [8]. In order to discover more potent and selective anticancer agents for further development and as a continuation of our studies, we aimed to investigate the structure activity relationship and carry out optimization of new indolyl-oxadiazoles.

In this paper, we further modified the connector (**X**) between the oxadiazole ring and an aromatic ring, to further investigate the role of the connecting group in the structural optimization with respect to fitting into the active site of the Bcl-2 protein (Figure 1).

## 2. Results

### 2.1. Synthesis of Target Compounds

**Scheme 1:**
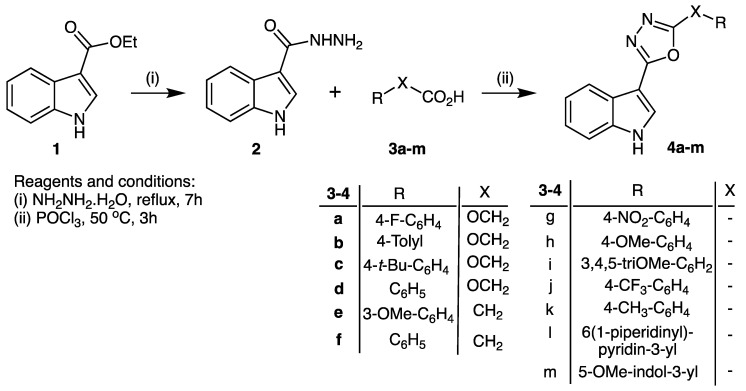
Synthesis of 2-(1*H*-indol-3-yl)-5-substituted-1,3,4-oxadiazoles **4a–m**.

Reaction of commercially available ethyl indol-3-yl carboxylate (**1**) with hydrazine hydrate under reflux gave the corresponding hydrazide (**2**) in 80% isolated yield, according to the previously reported method [5,6,7]. The intermediate hydrazide (**2**) was then reacted with aryl-linked carboxylic acids (**3a–m**) in the presence of phosphorus oxychloride (50 °C), to provide the product 2-(1H-indol-3-yl)-5-substituted-1,3,4-oxadiazoles (**4a–m**) in 46–66% yield (Scheme 1).

### 2.2. In Vitro Antiproliferative Activity

Evaluation of anti-proliferative activity for the new 2-(1*H*-indol-3-yl)-5-substituted-1,3,4-oxadiazoles (**4a–m**) was carried out in Bcl-2-expressing human metastatic breast (MDA-MB-231) [9] and cervical (HeLa) [10] cancer cell lines, using the MTT endpoint assay, following 72 h incubation of test compound, according to previously established protocols [5,6,7,8]. Further analysis within the non-adherent leukemic cell lines KG1a (acute myelogenous leukemia, Bcl-2 +ve) [11] and Jurkat (T-cell leukemia, Bcl-2 −ve,) [12] was carried out by using the CellTiter-Blue^®^ assay according to published precedent [5,6,7,8]. The results (IC_50_ values in mM, mean values from three independent experiments) are presented in Table 1.

With the exception of compounds **4a**, **4b** and **4f**, the test compounds were found to inhibit the growth of the Bcl-2 expressing cell lines (MDA-MB-231, HeLa and KG1a) with IC_50_ values < 10 μM. In general, the Bcl-2 negative cell line (Jurkat) was found to be unresponsive. Considering the different linker groups, the methylene linker (X = CH_2_) gave low potency values for compound **4e** and the unresponsive compound **4f**. The more active group of compounds appeared to be those where the oxadiazole ring was directly connected to the aryl group (no linker), with compounds **4j** (R = CF_3_Ph) and **4k** (R = 4-CH_3_-Ph) being particularly potent. A selection of these most active compounds plus compounds of intermediate activity were taken forward to study for competitive Bcl-2 binding in the ELISA assay.

### 2.3. Evaluation of Bcl-2 Binding (ELISA Assay)

The most active compounds from the cell line assay (**4j** and **4k**), along with the moderately active methyleneoxy linker compounds (**4c** and **4d**), the methylene linker compound **4e** and other active compounds with no linker (**4i** and **4m**), plus the inactive compound **4b**, were further studied. Compounds were evaluated according to their ability to compete with the pro-apoptotic BH3 domain Bim peptide for binding to His-tagged Bcl-2 protein, with a readout from anti-His secondary antibody conjugated to HRP (horseradish peroxidase) enzyme that produces a colorimetric readout in the presence of *o*-phenylenediamine (competitive ELISA assay). The protocol followed our previously published work on Bcl-2 inhibitors [5,6,7,8], and the results are shown in Table 2. The clinical trial stage Bcl-2 inhibitory natural product (-)-gossypol [13] was used as a positive control.

ELISA assay results largely mirrored the data obtained from Bcl-2 positive cancer cell lines. Again compounds **4j** and **4k** stood out as the most active at preventing Bim peptide binding, and notably were more potent than the standard positive control Bcl-2 inhibitor (−)-gossypol. Compounds with low-level cellular activity, such as **4b**, were inactive in the ELISA assay; however, compound **4i**, one of the most active in the cell line assay, had only moderate Bcl-2 binding activity according to the ELISA assay.

### 2.4. Molecular Modeling of Compound 4j within the Bcl-2 Binding Pocket

**Figure 2:**
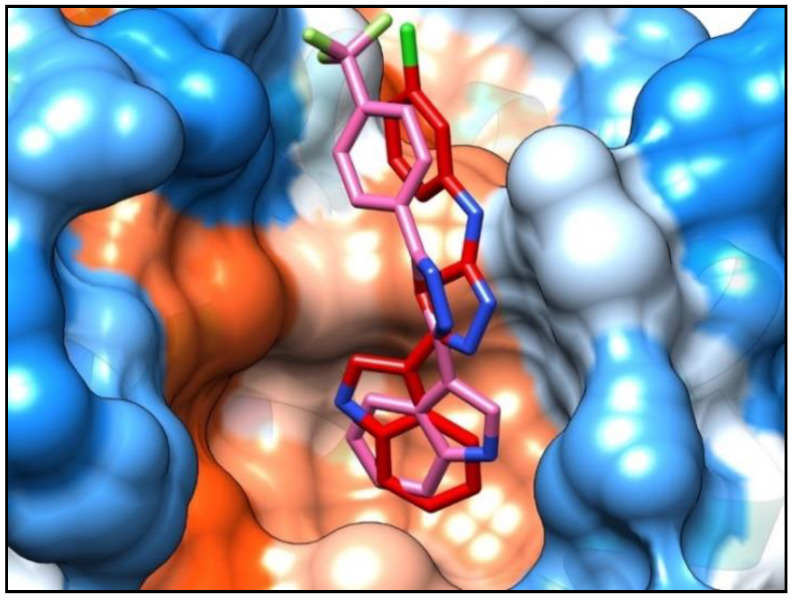
Compound **4j** (pink stick) showed a similar binding mode to our previous lead compound 5-(1*H*-indol-3-yl)-N-(3-chlorophenyl)-1,3,4-oxadiazol-2-amine [5] (red stick). Both compounds showed a good fit within the hydrophobic pocket of the Bcl-2 protein.

**Figure 3:**
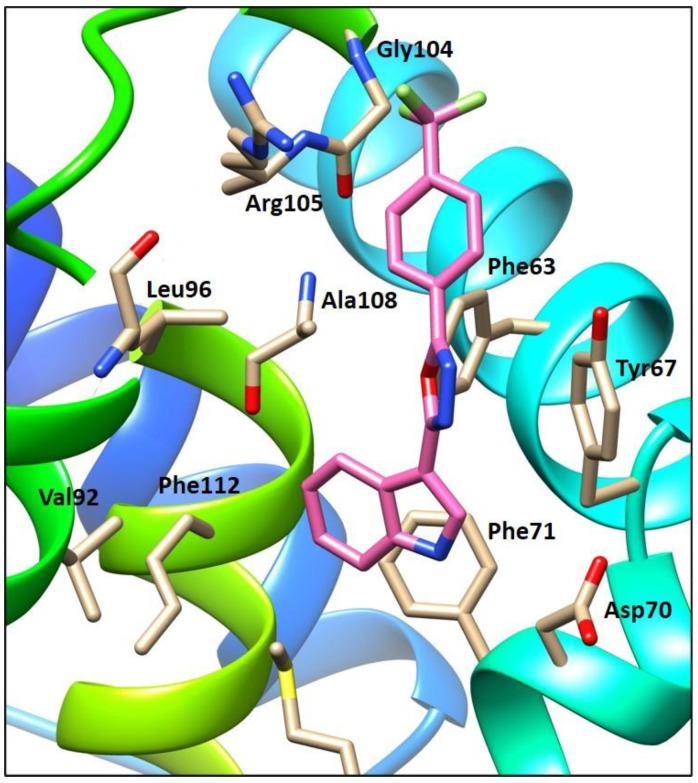
Compound **4j** showed hydrophobic interactions with key residues Tyr67, Phe71, Leu96 and Ala108, in addition to a p-cation interaction between the trifluorophenyl group and the key residue Arg105.

A molecular modeling study was performed to better understand and rationalize the potent cellular anticancer activity of the new indolyl-aryl-oxadiazole derivative **4j**, mediated by Bcl-2 inhibition. Docking studies were carried within MOE [14], using the published Bcl-2 crystal structure (PDB: 4AQ3) [15]. Docking of our previous lead compound 5-(1*H*-indol-3-yl)-N-(3-chlorophenyl)-1,3,4-oxadiazol-2-amine [5] and the most active compound **4j** of the new series of indolyl-oxadiazoles was carried out at the Bim peptide binding site. Results showed that the new derivative **4j**, like the previous hit compound, fits well within the hydrophobic pocket (Figure 2). Compound **4j** showed a p-cation interaction between the trifluorophenyl group and the key residue Arg105, and a hydrophobic interaction between the Bcl-2 hydrophobic groove residue Tyr67 and the oxadiazole moiety. Additional hydrophobic interactions were observed with Leu96 and Ala108 key residues. Compound **4j** showed a comparatively tighter binding energy score in comparison with our previous lead (−5.64 and −4.81 kcal, respectively), indicative of the good fit of compound **4j** within the binding pocket. Notably, the orientation of the indole ring of compound **4j** in Figure 2 is inverted compared to the corresponding indole ring of the previously reported lead compound [5]. Orientation of the indole ring of **4j** within the deep hydrophobic pocket allows additional interactions between the indole NH and the neighboring Asp70 carboxylate residue that are not present in the orientation of 5-(1*H*-indol-3-yl)-N-(3-chlorophenyl)-1,3,4-oxadiazol-2-amine [5], which might help explain the lower relative binding energy score for compound **4j**. Representations of the binding interactions of **4j** within the Bcl-2 hydrophobic pocket are shown in Figure 3.

### 2.5. Physicochemical Properties and Assessment of Hit Optimization Potential

Assessment of key drug-like properties was undertaken for the most active compounds (**4d**, **4e** and **4j**) from the three synthesized mini-series, representing each of the linker functions (Table 3). Calculated physicochemical data are also presented for the structurally related clinical drug candidate obatoclax. The key parameters of molecular weight (MW), calculated logP, topological polar surface area (TPSA), number of hydrogen bond acceptors (nON) and number of hydrogen bond donors (nOHNH) were calculated by using the freely available Molinspiration^®^ software [16].

Analysis of the drug-like properties of hit compounds reveals that **4d**, **4e** and **4j** all fall within the expected parameters for further development. Of particular note are the properties of the most potent analogue **4j**. Compound **4j** is rather lipophilic, with a calculated logP value of 4.77, just within the recommended Lipinski rule of 5 for prediction of oral bioavailability, and similar to the free base of the related clinical drug candidate obatoclax. This preliminary physicochemical data gives confidence for further development of compound **4j**, noting the possibility that a mesylate (or other) salt form may be required for optimization of solubility whilst maintaining potent cellular activity, as is the case for obatoclax.

## 3. Discussion

The results presented here report further evidence of the ability of synthetically accessible heterocycles based on the indolyl-oxadiazole scaffold to act as potent Bcl-2 inhibitory anticancer agents. This early drug discovery work, inspired by clinical stage Bcl-2 inhibitors such as obatoclax mesylate, has led to a new series of anticancer compounds obtained in two chemical steps from commercially available starting materials. New compounds were designed to test the optimal linker function between the 5-position of oxadiazole and a substituted aryl group, resulting in the discovery of 2-(1*H*-indol-3-yl)-5-(4-trifluoromethylphenyl)-1,3,4-oxadiazole (**4j**) as the most potent compound of the series. Compound **4j** (and several other examples) possessed low- to sub-micromolar IC_50_ values against Bcl-2 expressing human cancer cell lines such as MDA-MB-231 (invasive breast), HeLa (cervical) and KG1a (acute myelogenous leukemia). In addition, compound **4j** was able to compete with the pro-apoptotic BH3 domain Bim peptide for binding to His-tagged Bcl-2 protein in a competitive ELISA assay. Low micromolar IC_50_ values were obtained, but they were more potent than the positive control clinical stage compound (−)-gossypol. The Bcl-2 binding activity at the Bim peptide binding site was rationalized by computational modeling (MOE environment), offering a rational basis for further optimization to a lead Bcl-2 inhibitory anticancer agent. Finally, preliminary physicochemical properties of **4j** were compatible with the possibility of further development of this compound, most likely in salt form, to overcome the relatively high lipophilicity of this particular heterocycle.

## 4. Materials and Methods

### 4.1. General Experimental Details—Chemistry

All starting materials and solvents were used without further purification. Melting points were measured on a Griffin apparatus and are uncorrected. Nuclear magnetic resonance (NMR) spectra were recorded on a Bruker Avance 500 MHz instrument, and chemical shifts (δ values) are given in ppm relative to Me_4_Si, with coupling constants (*J* values) in Hz. Mass spectrometry was run in electrospray ionization (ESI) mode (Bruker MicroTof instrument, Coventry, UK). Elemental analysis (% CHN) was run by combustion analysis through an outsourced service (Medac Ltd., Surrey, UK).

### 4.2. Chemical Synthesis

#### 4.2.1. Synthesis of 3-Indolylcarboxlic Acid Hydrazide (2)

Excess hydrazine monohydrate (5 mL) was added to a solution of methyl indolyl-3-carboxylate (1.0 g, 10 mmol) in ethanol (10 mL). The reaction mixture was heated under reflux for 7 h and then left to cool to room temperature. The resulting precipitate was collected by filtration and washed with water, followed by cold ethanol, to remove excess hydrazine. After drying under vacuum, the corresponding acid hydrazide was obtained, and it was used without further purification. Yield 80%, m.p. 205–207 °C. ^1^H NMR (DMSO-*d_6_*) δ 4.30 (s, 2H, NH_2_), 7.107–7.14 (m, 2H, ArCH), 7.43 (d, 1H, *J* = 8.10, ArCH), 7.96 (s, 1H, ArCH), 8.14 (d, 1H, *J* = 8.14, ArCH), 9.13 (s, 1H, NH), 11.49 (s, 1H, NH indole).

#### 4.2.2. General Method for Synthesis of 2-(1H-Indol-3-Yl)-5-Substituted-1,3,4-Oxadiazoles (4a–m)

A mixture of the acid hydrazide **2** (10 mmol), carboxylic acid derivatives **3a–m** (10 mmol) and phosphorus oxychloride (5 mL) was heated for 4 h, at 50 °C, and then left to cool to room temperature. Ice was added, and the reaction mixture was stirred overnight; then the mixture was carefully neutralized with saturated sodium bicarbonate solution. The precipitate formed was filtered, dried and crystallized from ethanol, to give the corresponding oxadiazole product **4a–m**.

2-(1*H*-Indol-3-yl)-5-(4-fluorophenoxymethyl)-1,3,4-oxadiazole (**4a**). Yield 60%, m.p. 210–212 °C. ^1^H NMR (DMSO-*d_6_*): δ 5.45 (s, 2H, CH_2_), 7.107–7.16 (m, 4H, ArCH), 7.197–7.30 (m, 2H, ArCH) 7.53 (d, 1H, *J* = 7.5, ArCH-2, indole), 8.12 (d, 1H, *J* = 7.5, ArCH), 8.30 (d, 1H, J = 1.5, ArCH), 12.14 (s, 1H, NH indole). ^13^C-NMR (DMSO-*d_6_*): δ 60.18 (CH_2_), 99.01 (C-3), 112.36 (ArCH), 115.92 (ArCH), 121.26 (ArCH), 122.90 (ArCH), 124.04 (ArCH), 127.74 (ArCH), 128.52 (ArCH), 136.43 (ArC), 153.70 (ArC), 156.20 (ArC), 158.09 (ArC), 160.17 (CO), 162.68 (CO). Anal. (C_17_H_12_FN_3_O): % CHN required: C 66.02, H 3.91, N 13.59; found: C 65.62, H 3.85, N 13.58. MS (C_17_H_12_FN_3_O): calcd mass 309.09, found (*m*/*z*, ES^+^): 310.10.

2-(1*H*-Indol-3-yl)-5-(4-methylphenoxymethyl)-1,3,4-oxadiazole (**4b**). Yield 56%, m.p. 201–203 °C. ^1^H-NMR (DMSO-*d_6_*): δ 2.67 (s, 3H, CH_3_) 5.83 (s, 2H, CH_2_), 7.42 (s, 1H, ArCH), 7.55 (d, 2H, *J* = 7.0, ArCH), 7.66.7.69 (m, 2H, ArCH), 7.94 (d, 1H, *J* = 7, ArCH), 8.47 (d, 1H, *J* = 7, ArCH), 8.70 (s, 1H, ArCH), 12.50 (s, 1H, NH indole). ^13^C NMR (DMSO-*d_6_*): 20.05 (CH_3_), 59.64 (CH_2_), 99.06 (C-3), 112.45 (ArCH), 114.84 (ArCH), 120.11 (ArCH), 121.24 (ArCH), 122.87 (ArCH), 124.06 (ArCH), 128.49 (ArCH), 129.93 (ArC), 130.53 (ArC), 136.44 (ArC), 155.31 (ArC), 160.38 (CO), 162.64 (CO). Anal. (C_18_H_15_N_3_O_2_) % CHN required: C 70.81, H 4.95, N 13.76; found: C 70.62, H 4.87, N 13.85. MS (C_18_H_15_N_3_O_2_): calcd mass 305.33, found (*m*/*z*, ES^+^): 306.12.

2-(1*H*-Indol-3-yl)-5-(4-*tert*-butylphenoxymethyl)-1,3,4-oxadiazole (**4c**). Yield 51%, m.p. 218–220 °C. ^1^H NMR (DMSO-*d_6_*): δ 1.26 (s, 9H, t-butyl), 5.40 (s, 2H, CH_2_), 7.04 (d, 2H, *J* = 9, ArCH), 7.24–7.26 (m, 2H, ArCH), 7.34 (d, 2H, *J* = 9, ArCH), 7.54 (d, 1H, *J* = 8, ArCH), 8.08 (d, 1H, *J* = 8, ArCH), 8.20 (d, 1H, *J* = 3, ArCH), 12.96 (s, 1H, NH indole). ^13^C NMR (DMSO-*d_6_*): δ 31.24 (CH_3_), 33.81 (ArC), 59.57 (CH_2_), 99.05 (C3), 112.46 (ArCH), 114.43 (ArCH), 120.10 (ArCH), 121.24 (ArCH), 122.88 (ArCH), 124.06 (ArC), 126.23 (ArCH), 128.49 (ArCH), 136.44 (ArC), 143.96 (ArC), 155.16 (ArC), 160.41 (CO), 162.65(CO). Anal. (C_21_H_21_N_3_O_2_) % CHN required: C 72.60, H 6.09, N 12.10; found: C 72.67, H 6.03, N 12.05. MS (C_21_H_21_N_3_O_2_): calcd mass 347.41, found (*m*/*z*, EI^+^): 347.16.

2-(1*H*-Indol-3-yl)-5-(phenoxymethyl)-1,3,4-oxadiazole (**4d**). Yield 46%, m.p. 195–197 °C. ^1^H NMR (DMSO-*d_6_*): δ 4.30 (s, 2H, CH_2_), 7.237–7.25 (m, 2H, ArCH), 7.307–7.33 (m, 1H, ArCH), 7.377–7.39 (m, 4H, ArCH), 7.52 (d, 1H, *J* = 8, ArCH), 8.06 (d, 1H, *J* = 8, ArCH), 8.10 (d, 1H, *J* = 2.5, ArCH), 12.96 (s, 1H, NH indole). ^13^C NMR (DMSO-*d_6_*): δ 58.70 (CH_2_), 99.40 (C3), 112.37 (ArCH), 120.10 (ArCH), 121.09 (ArCH), 122.76 (ArCH), 124.04 (ArC), 127.12 (ArCH), 127.89 (ArCH), 128.72 (ArCH), 128.79 (ArCH), 134.87 (ArC), 136.39 (ArC), 162.05 (CO), 163.08 (CO). Anal. (C_17_H_13_N_3_O_2_): % CHN required: C 70.09, H 4.50, N 14.42, found: C 69.62, H 4.39, N 14.31.

2-(*1H*-Indol-3-yl)-5-(3-methoxybenzyl)-1,3,4-oxadiazole (**4e**). Yield 56%, m.p. 208–210 °C. ^1^H NMR (DMSO-*d_6_*): δ 3.84 (s, 3H, OCH_3_) 4.52 (s, 2H, CH_2_) 6.90–6.94 (m, 1H, ArCH), 7.15 (d, 1H, *J* = 7.7, ArCH), 7.22–7.26 (m, 2H, ArCH), 7.33–7.36 (m, 2H, ArCH), 7.55 (d, 1H, *J* = 8.5, ArCH), 8.12 (d, 1H, *J* = 6.5, ArCH), 8.17 (d, 1H, *J* = 2.3, ArCH), 11.98 (s, 1H, NH indole). ^13^C NMR (DMSO-*d_6_*): 25.54 (CH_2_), 55.51 (OCH_3_), 99.50 (C3), 111.10 (ArCH), 112.36 (ArCH), 120.09 (ArCH), 120.49 (ArCH), 121.04 (ArCH), 122.73 (ArCH), 124.06 (ArC), 127.72 (ArC), 128.81 (ArCH), 130.23 (ArCH), 136.38 (ArC), 157.03 (ArC), 161.79 (CO), 162.99 (CO). Anal. (C_18_H_15_N_3_O_2_) % CHN required: C 70.81, H 4.95, N 13.76, found: C 70.84, H 4.85, N 13.76. MS (C_18_H_15_N_3_O_2_): calcd mass 305.33, found (*m*/*z*, ES^+^): 306.12.

2-(1*H*-Indol-3-yl)-5-benzyl-1,3,4-oxadiazole (**4f**). Yield 47%, m.p. 187–189 °C. ^1^H NMR (DMSO-*d_6_*): δ 4.30 (s, 2H, CH_2_), 7.227–7.26 (m, 2H, ArCH), 7.31 (t, 1H, *J* = 7, ArCH), 7.367–7.39 (d, 4H, *J* = 8, ArCH), 7.52 (d, 1H, *J* = 8, ArCH), 8.06 (d, 1H, *J* = 7.60, ArCH), 8.11 (s, 1H, ArCH), 11.97 (s, 1H, NH indole). ^13^C NMR (DMSO-*d_6_*): δ 21.09 (CH_2_), 99.46 (C3), 112.44 (ArCH), 120.22 (ArCH), 120.98 (ArCH), 121.23 (ArCH), 122.83 (ArCH), 124.11 (ArCH), 126.28 (ArCH), 128.41 (ArCH), 129.89 (ArC), 136.46 (ArC), 141.58 (ArC), 161.72 (CO), 161.92 (CO). Anal. (C_17_H_13_N_3_O) % CHN required: C 74.17, H 4.76, N 15.26, found: C 74.16, H 4.70, N 15.24. MS (C_17_H_13_N_3_O): calcd mass 275.30, found (*m*/*z*, EI^+^) 275.10.

2-(1*H*-Indol-3-yl)-5-(4-nitrophenyl)-1,3,4-oxadiazole (**4g**). Yield 64%, m.p. 215–217 °C. ^1^H NMR (DMSO-*d_6_*) δ 7.277–7.30 (m, 2H, ArCH), 7.57 (d, 1H, *J* = 8.5, ArCH), 8.17 (d, 1H, *J* = 8.5, ArCH), 8.348–8.37 (m, 3H, ArCH), 8.53 (d, 2H, *J* = 9, ArCH), 12.18 (s, 1H, NH indole). ^13^C NMR (DMSO-*d_6_*): δ 99.06 (C3), 120.33 (ArCH), 122.95 (ArCH), 123.67 (ArCH), 124.07 (ArCH), 127.48 (ArCH), 127.71 (ArCH), 129.14 (ArCH), 129.23 (ArC), 130.65 (ArC), 148.76 (ArC), 159.72 (ArC), 160.56 (CO), 162.87 (CO). Anal. (C_16_H_10_N_4_O_3_) % CHN required: C 62.74, H 3.29, N 18.29, found: C 62.75, H 3.25, N 17.93. MS (C_16_H_10_N_4_O_3_): calcd mass 306.28, found (*m*/*z*, EI^+^): 306.

2-(1*H*-Indol-3-yl)-5-(4-methoxyphenyl)-1,3,4-oxadiazole (**4h**). Yield 64%, m.p. 210–212 °C. ^1^H NMR (DMSO-*d_6_*): δ 3.89 (s, 3H, OCH_3_) 7.12 (t, 1H, *J* = 6.5, ArCH), 7.21 (d, 2H, *J* = 9, ArCH), 7.28 (t, 1H, *J* = 7, ArCH), 7.31 (s, 1H, ArCH), 7.51 (d, 1H, *J* = 8.5, ArCH), 7.70 (d, 1H, *J* = 8, ArCH), 8.08 (d, 2H, *J* = 9, ArCH), 12.30 (s, 1H, NH indole). ^13^C NMR (DMSO-*d_6_*): δ 55.51 (OCH_3_), 99.50 (C3), 104.81 (ArCH), 112.21 (ArCH), 114.90 (ArCH), 115.57 (ArCH), 120.30 (ArCH), 121.19 (ArCH) 121.40 (ArC), 124.13 (ArCH), 127.72 (ArC), 137.76 (ArC), 158.90 (ArC), 162.10 (CO), 163.30 (CO). Anal. (C_17_H_13_N_3_O_2_) % CHN required: C 70.09, H 4.50, N 14.42, found: C 69.82, H 4.40, N 14.25. MS (C_17_H_13_N_3_O_2_): calcd mass 291.30, found (*m*/*z*, EI^+^): 291.00.

2-(1*H*-Indol-3-yl)-5-(3,4,5-trimethoxyphenyl)-1,3,4-oxadiazole (**4i**). Yield 60%, m.p. 221–222 °C. ^1^H NMR (DMSO-*d_6_*): δ 3.83 (s, 3H, OCH_3_), 3.92 (s, 3H, OCH_3_), 3.99 (s, 3H, OCH_3_), 6.90 (s, 1H, ArCH), 7.27–7.30 (m, 2H, ArCH), 7.49 (s, 1H, ArCH), 7.56 (t, 1H, *J* = 7.5, ArCH), 8.18 (t, 1H, *J* = 6, ArCH), 8.23 (d, 1H, *J* = 3, ArCH), 12.01 (s, 1H, NH indole). ^13^C NMR (DMSO-*d_6_*): δ 55.88 (OCH_3_), 56.21 (OCH_3_), 56.82 (OCH_3_), 98.72 (ArCH), 98.86 (C3), 103.52 (ArCH), 112.41 (ArCH), 120.16 (ArCH), 121.12 (ArCH), 122.74 (ArCH), 124.18 (ArC), 127.90 (ArC), 136.45 (ArC), 142.85 (ArC), 152.67 (ArC), 160.75 (CO), 161.34 (CO). Anal. (C_19_H_17_N_3_O_4_) % CHN required: C 64.95, H 4.88, N 11.96, found: C 64.94, H 4.75, N 12.00. MS (C_19_H_17_N_3_O_4_): calcd mass 351.36, found (*m*/*z*, EI^+^): 351.11.

2-(1*H*-Indol-3-yl)-5-(4-trifluoromethylphenyl)-1,3,4-oxadiazole (**4j**). Yield 63%, m.p. 224–226 °C. ^1^H NMR (DMSO-*d_6_*): δ 7.267–7.30 (m, 2H, ArCH), 7.58 (d, 1H, *J* = 8.5, ArCH), 8.0 (d, 2H, *J* = 8.5, ArCH), 8.18 (d, 1H, *J* = 7, ArCH), 8.34 (d, 2H, *J* = 8, CH aromatic), 8.37 (d, 1H, J = 3, ArCH), 12.06 (s, 1H, NH indole). ^13^C NMR (DMSO-*d_6_*): δ 99.15 (C3), 112.52 (ArCH), 121.38 (ArCH), 122.95 (ArCH), 126.35 (ArCH), 126.38 (ArCH), 127.13 (ArC), 127.48 (ArCH), 128.99 (ArCH), 130.97 (ArC), 131.23 (ArC), 136.49 (ArC), 160.88 (CO), 162.58 (CO). Anal. (C_17_H_10_F_3_N_3_O) % CHN required: C 62.01, H 3.06, N 12.76, found: C 62.10, H 3.13, N 12.75. MS (C_17_H_10_F_3_N_3_O): calcd mass 329.00, found (*m*/*z*, EI^+^): 329.08.

2-(1*H*-Indol-3-yl)-5-(4-methylphenyl)-1,3,4-oxadiazole (**4k**). Yield 66%, m.p. 198–200 °C. ^1^H NMR (DMSO-*d_6_*): δ 2.43 (s, 3H, CH_3_), 7.29 (dd, 2H, *J* = 6.3, 2.6, ArCH), 7.45 (d, 2H, *J* = 7, ArCH), 7.56 (d, 1H, *J* = 6.5, ArCH), 8.02 (d, 2H, *J* = 8, ArCH), 8.18 (d, 1H, *J* = 6.5, ArCH), 8.30 (s, 1H, ArCH), 12.06 (s, 1H, NH indole). ^13^C NMR (DMSO-*d_6_*): δ 21.10 (CH_3_), 99.45 (C3), 112.44 (ArCH), 120.22 (ArCH), 120.99 (ArCH), 121.23 (ArCH), 122.83 (ArCH), 124.11 (ArCH), 126.29 (ArCH), 128.43 (ArC), 129.90 (ArC), 136.46 (ArC), 141.59 (ArC), 161.72 (CO), 161.92 (CO). Anal. (C_17_H_13_N_3_O) % CHN required: C 74.17, H 4.76, N 15.26, found: C 73.97, H 4.59, N 15.25. MS (C_17_H_13_N_3_O): calcd mass 275.30, found (*m*/*z*, ES^−^): 274.10.

2-(1*H*-Indol-3-yl)-5-(6-(piperidin-1-yl)-pyridin-3-yl)-1,3,4-oxadiazole (**4l**). Yield: 53%, m.p. 240–242 °C. ^1^H NMR (DMSO-*d_6_*): δ 1.56 (d, 4H, *J* = 4, CH aliphatic), 1.66 (d, 2H, *J* = 5, CH aliphatic), 3.663–3.69 (m, 4H, CH aliphatic), 7.01 (d, 1H, *J* = 9, ArCH), 7.247–7.30 (m, 2H, ArCH), 7.55 (d, 1H, *J* = 8, ArCH), 8.10 (dd, 1H, *J* = 9, ArCH), 8.16 (d, 1H, *J* = 6.5, ArCH), 8.26 (d, 1H, *J* = 3, ArCH), 8.80 (d, 1H, *J* = 2.5, ArCH), 12.02 (s, 1H, NH indole). ^13^C NMR (DMSO-*d_6_*): δ 24.18 (CH_2_), 25.09 (CH_2_), 45.23 (CH_2_), 99.60 (C3), 106.42 (ArCH), 107.81 (ArCH), 112.38 (ArCH), 120.26 (ArCH), 121.14 (ArCH), 122.78 (ArCH), 124.08 (ArC), 128.11 (ArCH), 135.09 (ArC), 136.44 (ArC), 146.62 (ArC), 159.35 (ArC), 160.88 (CO), 160.95 (CO). Anal. (C_20_H_19_N_5_O) % CHN required: C 69.55, H 5.54, N 20.28, found: C 69.26, H 5.37, N 20.18. MS (C_20_H_19_N_5_O): calcd mass 345.40, found (*m*/*z*, EI^+^): 345.16.

2-(1*H*-Indol-3-yl)-5-(5-methoxy-1*H*-indol-2-yl)-1,3,4-oxadiazole (**4m**). Yield 63%, m.p. 231–233 °C. ^1^H NMR (DMSO-*d_6_*): δ 3.80 (s, 3H, OCH_3_), 6.93 (dd, 1H, *J* = 8, ArCH), 7.177–7.19 (m, 2H, *J* = 2.5, ArCH), 7.267–7.32 (m, 2H, ArCH), 7.41 (d, 1H, *J* = 9, ArCH), 7.55-7.58 (m, 1H, ArCH), 8.21-8.23 (m, 1H, ArCH), 8.27 (s, 1H, ArCH), 12.09 (s, 2H, NH indole). ^13^C NMR (DMSO-*d_6_*): δ 55.28 (OCH_3_), 99.30 (C3), 101.90 (C2), 104.02 (ArCH), 112.48 (ArCH), 112.97 (ArCH), 115.03 (ArCH), 120.28 (ArCH), 121.27 (ArCH), 121.75 (ArCH), 122.90 (ArCH), 124.08 (ArC), 127.80 (ArC), 128.37 (ArC), 132.96 (ArC), 136.46 (ArC), 154.09 (ArC), 157.30 (CO), 161.26 (CO). Anal. (C_19_H_14_N_4_O_2_) % CHN required: C 69.08, H 4.27, N 16.96, found: C 68.89, H 4.14, N 16.90. MS (C_19_H_14_N_4_O_2_): calcd mass 330.34, found (*m*/*z*, EI^+^): 330.10.

### 4.3. Cell Viability—MTT Assay (MDA-MB-231 and HeLa Cells)

Human breast cancer (MDA-MB-231) and cervical cancer (HeLa) cells, obtained from the ATCC (Manassas, VA, USA), were cultured and maintained as previously described [5,6,7,8]. For each experiment, 3000 cells in 0.2 mL of medium were seeded into each well of a clear flat-bottomed 96-well plate and allowed to adhere for 24 h. The cells were then incubated with the test compound (from 10 mM stock in DMSO), over a 10-fold dilution series, with concentrations ranging from 0.00001 to 100 μM and each diluent being performed in triplicate. Cells were incubated with the test compound for 72 h, followed by treatment with 3-[4,5-dimethylthiazol-2-yl]-2,5-diphenyl tetrazolium bromide (MTT) solution and reading of absorbance (automated microplate) according to our previous protocol [5,6,7,8]. Mean IC_50_ values were obtained by using GraphPad Prism 5 software (San Diego, CA, USA). For each concentration, three independent repeat experiments were carried out to establish reproducibility.

### 4.4. Cell Viability—CellTiter-Blue^®^ Assay (KG1a and Jurkat Cells)

Human acute myeloid leukemia KG1a and acute T-cell lymphocytic Jurkat cells were cultured and maintained as previously described [5,6,7,8]. For each experiment 15,000 cells in 0.1 mL of medium were seeded into each well of a solid-black fluorescence 96-well plate and incubated for 24 h. The cells were then incubated with test compound (from 10 mM stock in DMSO), over a 10-fold dilution series, with concentrations ranging from 0.00001 to 100 μM and each diluent being performed in triplicate. Cells were incubated with test compound for 24 h, followed by treatment with CellTiter-Blue^®^ solution and reading of fluorescence according to our previous protocol [5,6,7,8]. Mean IC_50_ values were obtained by using GraphPad Prism 5 software (San Diego, CA, USA). For each concentration, three independent repeat experiments were carried out, to establish reproducibility.

### 4.5. Enzyme-Linked Immunosorbent Assay (ELISA)

The ELISA assay to determine the ability of test compounds (10-fold dilution series) to compete with the interaction between biotinylated Bim peptide and His-tagged Bcl-2 protein was carried out according to our previously published protocol [5,6,7,8]. The addition of anti-His antibody containing horseradish peroxidase enzyme, followed by the addition of *o*-phenylenediamine and hydrogen peroxide, allowed for the determination of optical density, using a plate reader (450 nm). The experiments were carried out on three separate occasions, including both negative and positive controls. A plot of log μM concentration for each inhibitor against the percentage reduction in affinity for the Bim peptide was created, using non-linear regression curve analysis (GraphPad Prism 5, San Diego, CA, USA) and the software used to generate the IC_50_ value for the Bcl-2 inhibitor.

### 4.6. Molecular Modeling and Docking

Bcl-2 protein (PDB:4AQ3) was prepared by using the MOE program (Chemical Computing Group, Cambridge, UK), ligands were built and the system was energy minimized by using the MMFF94x force field until a RMSD gradient of 0.05 Kcal mol^−1^ Å^−1^ was reached [14,15]. Triangle Matcher was chosen as the replacement methodology, and London dG was applied as the scoring function.

## 5. Conclusions

The work reported herein describes the design and synthesis of a new series of 2-(1*H*-indol-3-yl)-5-substituted-1,3,4-oxadiazoles (**4a–m**) as inhibitors of Bcl-2. Target compounds were readily synthesized through a cyclization reaction of indole carboxylic acid hydrazide **2** with substituted carboxylic acid derivatives **3a–m** in the presence of phosphorus oxychloride. Compound **4j**, containing a 4-trifluoromethylphenyl group directly attached to the oxadiazole ring, possessed the most potent anticancer activity across the Bcl-2 expressing human cancer cell lines (MDA-MB-231, HeLa, KG1a; sub-micromolar IC_50_ values). Compound **4j** displayed similarly potent activity in a Bcl-2 ELISA binding assay. Results were rationalized by computational docking within the Bcl-2 binding pocket, and compound **4j** (and other hit compounds) possessed satisfactory calculated early physicochemical (ADME) data for further development as a lead compound.

## Figures and Tables

**Table 1:** In vitro anti-proliferative activity of 2-(1H-indol-3-yl)-5-substituted-1,3,4-oxadiazoles **4a–m** in the MDA-MB-231 (metastatic breast), HeLa (cervical), KG1a (AML) and Jurkat (T-cell leukemia) human cancer cell lines.

Compound	X	R	IC_50_ (μM) ^1^			
			MDA-MB-231^2^	HeLa ^2^	KG1a ^3^	Jurkat ^3^
**4a**	OCH_2_	4-F-C_6_H_4_	36.11 ± 3.4	12.86 ± 1.33	>100	>100
**4b**	OCH_2_	4-CH_3_-C_6_H_4_	31.53 ± 4.8	13.31 ± 0.32	>100	>100
**4c**	OCH_2_	4-*t*Bu-C_6_H_4_	3.50 ± 0.16	0.9 ± 0.03	3.45 ± 0.8	>100
**4d**	OCH_2_	C_6_H_5_	1.63 ± 0.08	0.57 ± 0.04	2.56 ± 0.07	>100
**4e**	CH_2_	3-OMe-C_6_H_4_	6.28 ± 0.20	3.14 ± 0.14	3.66 ± 0.15	>100
**4f**	CH_2_	C_6_H_5_	>100	>100	>100	>100
**4g**	-	4-NO_2_-C_6_H_4_	6.95 ± 0.5	1.60 ± 0.12	>100	>100
**4h**	-	4-OMe-C_6_H_4_	6.02 ± 0.12	0.92 ± 0.08	2.21 ± 0.1	60.59 ± 0.12
**4i**	-	3,4,5-triOMe- C_6_H_2_	1.19 ± 0.1	1.22 ± 0.9	0.975 ± 0.02	12.25 ± 1.13
**4j**	-	4-CF_3_-C_6_H_4_	0.52 ± 0.15	0.88 ± 0.16	0.73 ± 0.09	>100
**4k**	-	4-CH_3_-C_6_H_4_	0.92 ± 0.19	1.28 ± 0.35	1.47 ± 0.13	>100
**4l**	-	6(1-piperidinyl)-pyridin-3-yl	2.97 ± 0.06	1.52 ± 0.03	4.5 ± 0.31	61.4 ± 1.4
**4m**	-	5-OMe-indol-3-yl	1.79 ± 0.07	1.19 ± 0.4	1.87 ± 0.14	57.45 ± 0.58

^1^ Mean values of three independent experiments (±SEM); ^2^ MTT assay; ^3^ CellTitre-Blue^®^ assay.

**Table 2:** ELISA binding activity IC_50_ values for selected 2-(1*H*-indol-3-yl)-5-substituted-1,3,4-oxadiazoles compared to positive control (-)-gossypol.

Compound	ELISA IC_50_ (µM) ^1^
**4b**	>100
**4c**	1.17 ± 0.13
**4d**	1.41 ± 0.01
**4e**	2.72 ± 0.2
**4i**	21.05 ± 1.09
**4j**	0.33 ± 0.05
**4k**	0.43 ± 0.05
**4m**	0.84 ± 0.05
**Gossypol**	0.60 ± 0.09

^1^ Mean of three independent experiments, with range quoted. Results are expressed as mean values ± SEM across three independent experiments.

**Table 3:** Analysis of physicochemical properties of hit compounds exhibiting Bcl-2 inhibitory activity (calculated by using software available at www.molinspiration.com).

Compound	X	R	MW	clogP	TPSA/Å	nON	nOHNH
**4d**	OCH_2_	C_6_H_5_	291.31	3.16	63.95	5	1
**4e**	CH_2_	3-OMe-C_6_H_4_	305.34	3.50	63.95	5	1
**4j**	-	4-CF_3_-C_6_H_4_	329.28	4.77	54.72	4	1
**Obatoclax**	-	-	317.39	4.08	53.71	4	2
